# Body Dissatisfaction, Cognitive Distraction, and Sexual Satisfaction in a Sample of LGB+ People: A Mediation Study Framed by Cognitive Psychology Models of Sexual Response

**DOI:** 10.3390/healthcare11222930

**Published:** 2023-11-09

**Authors:** Andreia A. Manão, Patrícia M. Pascoal

**Affiliations:** HEI-Lab: Digital Human-Environment Interaction Labs, Lusófona University, Campo Grande 376, 1749-024 Lisbon, Portugal; andreia.manao@ulusofona.pt

**Keywords:** sexual satisfaction, cognitive distraction, body dissatisfaction, cognitive models of sexual response, LGB+ people, sexual problems, sexual health

## Abstract

**Introduction:** Body dissatisfaction is a well-established risk factor for emotional problems and low levels of well-being indicators, such as sexual health. Cognitive models propose that dissatisfaction with one’s body can cause cognitive distraction related to physical appearance during sexual activity. This may compromise sexual response, namely, sexual satisfaction in heterosexual cis women. However, this relationship has only been studied within heterosexual samples. The present study aims to test a mediation model using cognitive distraction related to body appearance during sexual activity as a mediator between body dissatisfaction and sexual satisfaction in LGB+ cis people (lesbian, gay, bisexual, and other minority sexual orientations). **Methods:** This cross-sectional online study comprised 165 cisgender LGB+ participants (*n* = 67 cis women, 40.6%; *n* = 98 cis men, 59.4%). Self-report questionnaires were used: the Global Body Dissatisfaction Scale, the Body Appearance Distraction Scale, and a Single-Item Measure of Sexual Satisfaction. **Results:** Cis women and cis men experience similar levels of body dissatisfaction, cognitive distraction with body appearance during sexual activity, and sexual satisfaction. Body appearance cognitive distraction during sexual activity mediated the relationship between body dissatisfaction and sexual satisfaction only in the men’s sample. **Discussion:** Overall, in terms of gender and body dissatisfaction, our results reveal a reversed pattern than those found in heterosexual samples. This may be because LGB+ cis women may conform less to societal pressure, leading to less meaning given to body dissatisfaction in relation to sexuality, which may lead to more positive sexual outcomes. Likewise, LGB+ cis men present higher body dissatisfaction and experience lower sexual satisfaction, possibly due to the emphasis on physical appearance in the gay subculture. The results confirm the validity of cognitive models of sexual response.

## 1. Introduction

Body image is a multidimensional concept, and one of its dimensions is body dissatisfaction [1], a well-established risk factor for high levels of emotional problems (e.g., eating disorders [2]) and low levels of well-being indicators, especially in women [3]. Even though research indicates that societal pressure to attain an ideal body impacts men [4], it is widely agreed that these pressures are stronger for women of all ages, pressuring them to attain an ideal female body [5], and this translates into high levels of body dissatisfaction in women.

Initially formulated for cis women, Objectification Theory provides valuable insights into the underlying mechanisms of how societal pressures contribute to body dissatisfaction and its negative consequences [6,7]. This theory explains how exposure to sexual objectification (the idea that one’s global value derives from one’s sexual attractiveness according to shared standards of beauty) leads people to internalize cultural standards of attractiveness and to self-monitor their bodies to evaluate if they fit into these standards, which are associated with personal success. Failure to meet these standards can result in body shame and negative consequences such as impaired cognitive abilities, eating disorder symptoms, and depression [8]. According to this theory, women commonly internalize body dissatisfaction as the norm. In other words, most women feel dissatisfied with their bodies because of societal pressures, cultural norms, and media depictions of the “perfect” body. They are, therefore, at risk for emotional problems, namely, sexual problems that usually occur in a face-to-face context where the body is exposed and may be the object of other people’s evaluation.

Studies have shown that how people view their bodies is crucial to expressing their sexuality (e.g., [9]). A positive body image can foster a healthier and more authentic sexual identity, while a negative body image might lead to inhibitions and emotional distress within sexual contexts. In this line of reasoning, research has shown that feeling dissatisfied with one’s body is associated with less sexual satisfaction [10], which is an indicator of sexual health. More specifically, having negative thoughts and actions or experiencing negative emotions toward their body may lead to decreased satisfaction with one’s sex life. This is important because sexual satisfaction is crucial for overall well-being and life satisfaction [10]. This life and relationship satisfaction indicator is determined by mutual enjoyment and pleasure between partners in heterosexual samples [11].

A negative body image can significantly disrupt sexual function [12,13]. This impact can be attributed, in part, to the cognitive process of body appearance cognitive distraction during sexual activity, as suggested by the current cognitive models of sexual response [14,15]. Research framed by these models has shown that body dissatisfaction and focusing on specific body parts can lead to cognitive distraction during sexual activity for both cis men and cis women [12,14,16] and impairs a positive sexual response because cognitive distraction compromises the focus on positive sexual experiences and sensations [17]. More specifically, sexual dysfunctions can be better understood through cognitive models, particularly when considering the role of cognitive distraction. The term cognitive distraction originates in the concept of “spectatoring” introduced by Masters and Johnson [18]. “Spectatoring” refers to the intense self-focus during sexual interactions, potentially leading to sexual problems by increasing anxiety and distracting attention away from the erotic experience. After that, Barlow proposed a model that places cognitive distraction during sexual activities as a central element of erectile dysfunction [19]. This model was further supported by the Nobre’s Model [20,21]. It is also important to note that cognitive distraction differs from cognitive distortion. On one hand, cognitive distraction refers to a brief shift of attention away from an experience due to distracting thoughts or stimuli [22]. On the other hand, cognitive distortion involves more permanent and distorted thought patterns that can affect one’s perception of reality [23]. In the context of sexual activity, cognitive distraction involves having worries or self-consciousness about one’s body, causing a momentary lapse in concentration. 

Regardless of the thought’s content, the frequency of these thoughts is associated with sexual problems, highlighting the importance of considering attentional factors to understand the development and persistence of sexual problems [24]. Concerns related to performance and body image seem familiar in the context of sexual activity [24], and they may relate to preexisting predisposing factors. For example, pre-existing dissatisfaction with one’s body or specific body part(s) [13] may predispose one to engage in cognitive distraction related to one’s body appearance during sexual activity, disrupting the focus on the sexual cues and sensations. The literature also shows that this occurs in the general community and clinical samples of women and men with sexual dysfunction [14,16], placing body dissatisfaction as one crucial risk factor for cognitive distraction related to body appearance during sexual activity, which is the focus of the current study. 

LGB+ people (lesbian, gay, bisexual, and other minority sexual orientations) face unique experiences in a heteronormative and heterosexist culture [25]. Considering the social-based Minority Stress Model [25], LGB+ people experience several chronic stressors in a continuum from distal (i.e., prejudice-related events, external and objective) to proximal (i.e., internal and subjective events as a response to distal stressors). Discrimination and violence are some of the distal stressors, while internalized stigma and expectations of rejection are some of the proximal stressors. These stressors may impact different health outcomes, namely, mental health [26] and sexual well-being indicators, such as sexual satisfaction [27]. Existing research about sexual satisfaction has demonstrated that sexual satisfaction levels are similar in heterosexual women and men [28]. We did not find research that directly compares the levels of sexual satisfaction of cis women and cis men in LGB+ samples. Still, we found studies that compare heterosexual and LGB+ samples that demonstrated that lesbian women and gay men tend to present higher levels of sexual satisfaction than heterosexual women and heterosexual men [29,30]. This could happen due to the influence of different factors on the sexual satisfaction of LGB+ people [31] compared to heterosexual peers. For instance, body image among LGB+ people is often linked to minority stress [27], which can affect sexual satisfaction [32].

Studies have found that a more significant experience of sexual minority stressors [25] is associated with high body image concern levels [33]. A systematic review has demonstrated a significant correlation between sexual minority stress and body image concerns in women who identify as LGB+ [34]. However, research shows that the relationship between gender and body dissatisfaction differs for people who identify as LGB+ compared to those who identify as heterosexual. Specifically, heterosexual cis women are more likely to experience body dissatisfaction, in a similar way to bisexual or gay men, and LGB+ women are generally less prone to experience body dissatisfaction, similarly to heterosexual men [35,36].

To our knowledge, even though there is research on body dissatisfaction and cognitive distraction in LGB+ people [32,37], studies on the link between body dissatisfaction and cognitive distraction during sexual activity that use a comprehensive cognitive model of sexual outcomes have been limited to samples of heterosexual people [14,38]. Additionally, positive outcomes such as sexual satisfaction have not been considered in these investigations. It is crucial to expand our knowledge of the effects of cognitive processes on different outcomes and groups. Since research has shown that there are health disparities between LGB+ people and heterosexual people regarding various health behaviors and conditions [25], studying these factors may help identify ways to protect LGB+ people from these disparities that translate to or are related to negative outcomes. Regarding trans people, it is essential to acknowledge that the reasons for body dissatisfaction among trans people differ from those experienced by cisgender people [39]. This distinction is crucial as it emphasizes the specific factors that contribute to body dissatisfaction in trans people. Trans people may not be eligible subjects for studies solely focused on cisgender bodies, underlining the need for research to address their unique experiences.

**Figure 1:**
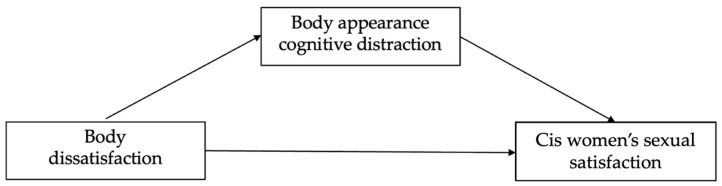
The mediator effect of cognitive distraction based on body appearance in the relationship between body dissatisfaction and cis women’s sexual satisfaction.

**Figure 2:**
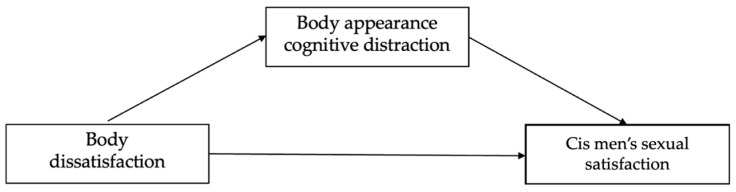
The mediator effect of cognitive distraction based on body appearance in the relationship between body dissatisfaction and cis men’s sexual satisfaction.

This paper aimed to fill the gap in the current literature regarding cognitive processes and factors that are related to the specificities of sexual satisfaction in LGB+ people by examining the mediating role of body appearance cognitive distraction between body dissatisfaction and sexual satisfaction in cis women and cis men who self-identify as LGB+ in the context of the cognitive model of sexual response (Figure 1 and Figure 2). Based on this model and related empirical findings, Objectification Theory, and Minority Stress Theory, we propose the following hypothesis that is formulated concerning gender status (cis women or cis men): cis women present higher body dissatisfaction and body appearance cognitive distraction levels when compared with cis men (H1); body dissatisfaction presents significant and negative correlations with sexual satisfaction in both samples (H2); and cognitive distraction mediates the relationship between body dissatisfaction and sexual satisfaction (H3).

## 2. Materials and Methods

### 2.1. Participants and Procedure 

A total of 165 cisgender LGB+ people were eligible for the current cross-sectional online quantitative study. It is impossible to determine the number of people reached and, among these, how many read the invitation to participate and decided not to participate since the study was advertised through online social media following a snowball-like procedure, which results in an unknown compliance rate. 

This study is part of a larger cross-sectional project developed online aimed at studying transdiagnostic factors associated with sexual outcomes. The inclusion criteria were being over 18 years of age (age of consent), mastering the Portuguese language, having had face-to-face sexual interaction with another person, and self-identifying as cis-gendered. The study received ethical approval from the relevant ethics committee. After hosting the study on a secure platform (Qualtrics, Provo, UT, USA), we tested the online protocol for length, ease of understanding, and flow with a group of students. The study’s data were collected online using different dissemination methods: convenience sampling (i.e., advertising online social networks) and snowball-like sampling (i.e., the participants referred the study to other potential participants). The first page online provided participants with a detailed explanation of the study’s goals, the criteria for eligibility, and the voluntary nature of their participation. No incentives were offered, and they could stop at any moment without any consequence. The email from the principal investigator was provided in case the participants needed to address relevant questions that led to complete information about the study. Participants were informed about the confidentiality of their participation, namely that the IP numbers and geolocation data were deleted and that the database was protected with a password only accessible to the research team. For the current study, we selected those participants who self-identified as LGB+ and answered that they had had face-to-face sexual activity. Sexual activity was defined in line with the measure used to assess cognitive distraction. This measure was adapted to include diverse sexual practices: “Sexual activity refers to mutual stimulation of genitals, oral sex, anal sex, intercourse, and other forms of face-to-face sexual stimulation” [22]. Data were collected between December 2019 and March 2020.

### 2.2. Measures

**The Demographic Overview Questionnaire** was developed for this study to assess sociodemographic data (e.g., age, area of residence, relationship status) and questions related to sexual issues (e.g., sexual orientation, having or having already had psychological or sexology treatment).

**The Global Body Dissatisfaction Scale** (GBD) [40] is a subscale of the Body Attitudes Test. It measures, with 4 items, how often a person has negative thoughts, behaviors, and feelings about their body. It is answered using a Likert scale from 0 (never) to 5 (always), with higher scores indicating higher body dissatisfaction levels. Participants’ answers ranged from 0 to 5 in the 4 items. In a sample of students without eating disorders, the mean score for this subscale was 6.62. The mean score for samples of people with various eating disorders was greater than 11 [41]. The reliability and validity of this measure were found to be high in Portuguese samples, with Cronbach’s alphas higher than 0.70 [14,16]. In the current study, the scale showed good reliability values both in the sample of cis women (α = 0.87) and in the sample of cis men (α = 0.79). Cronbach’s alpha for the total sample is 0.94.

**The Body Appearance Cognitive Distraction Scale** (BACDS) [22] is a subscale of the Cognitive Distraction Scale that evaluates how often people focus on their appearance during sexual involvement. It has 10 items, and in the current study, the original answering scale was inverted from 1 (never) to 6 (always) to make interpretation more intuitive, with higher scores reflecting higher cognitive distraction levels. Participants’ answers ranged from 1 to 6 on most items. The original study of BACDS indicated excellent consistency with a Cronbach’s alpha of 0.95 [22]. Additional studies in Portugal verified good reliability values with a Cronbach’s alpha exceeding 0.80 [14]. In the current study, the scale showed excellent reliability values both in the sample of cis women (α = 0.95) and in the sample of cis men (α = 0.93). Cronbach’s alpha for the total sample is 0.83.

**A Single-Item Measure of Sexual Satisfaction** was used to assess sexual satisfaction levels. The participants answered the question: “Over the past two months, how sexually satisfied have you been?” with a Likert scale from 1 (very satisfied) to 6 (not satisfied at all). Higher scores indicate less sexual satisfaction. The participants’ answers ranged from 1 to 6. Existing research that compared a single-item measure of sexual satisfaction with commonly used scales for assessing sexual satisfaction indicated that the single-item is adequate for research purposes, and its use has advantages, mainly due to the economy of measures [42].

### 2.3. Data Analysis

Data were analyzed in the IBM SPSS, Version 26 [43] and PROCESS MACRO for SPSS ([44]—Model 4). A one-way MANOVA (multivariate analysis of variance) was used to compare the total scores between cis women and cis men on the variables of interest. The parametric testing’s normality assumption was met since the skewness values were above |3| and the kurtosis scores above |10| were considered severe violations of normal distribution [45,46]. The relationship’s strength was interpreted using Cohen’s guidelines [47]. An effect size less than 0.29 is considered weak, between 0.30 and 0.49 is moderate, and above 0.50 is strong. A mediation model was used to determine whether the effect of body dissatisfaction on sexual satisfaction is mediated by cognitive distraction with the body’s appearance in two samples (cis women’s and cis men’s). Mediation analysis was used since it is a method of testing a theoretical causal chain wherein the independent variable (i.e., body dissatisfaction) has an impact on the mediator (i.e., cognitive distraction based on body appearance during sexual activity), which then influences the dependent variable (i.e., sexual satisfaction). The mediator serves to elucidate the causal connection between the two variables or, in other words, in the current study, we hypothesize that it explains a possible process by which the relationship between body dissatisfaction and sexual satisfaction can be explained. All CIs that excluded 0 were considered significant [48]. A significance level of *p* = 0.05 was used to test all the hypotheses. Participants with missing data in our quantitative variables of interest were removed from the quantitative analysis.

## 3. Results

This study’s sample comprised 165 cisgender LGB+ participants (*n* = 67 cis women, 40.6%; *n* = 98 cis men, 59.4%). Participants had a mean age of 32 (*SD* = 8.4), ranging from 18 to 55 years old. Seventy-three participants self-identified as exclusively homosexual (44.2%), 44 as preferably homosexual (26.7%), and 48 as bisexual (29.1%). Most of the participants were in a loving relationship (*n* = 65, 39.4%), 5 were married (3%), 43 were in a civil union/cohabitation (26.1%), 2 were divorced (1.2%), and 50 were single (30.3%). Most participants live in the Lisbon area (*n* = 101, 61.2%), 18 in the North (10.9%), 23 in the Center (13.9%), 8 in the Algarve (4.8%), 4 in the Alentejo (2.4%), 1 from the Autonomous Region of the Azores (0.6%), and 10 abroad (6.1%). All participants for the current analysis had had face-to-face sexual interaction with another person in the last 6 months. 

### 3.1. Group Comparison

A MANOVA was performed to investigate gender differences in the study’s variables. Three dependent variables were used: body dissatisfaction, body appearance, cognitive distraction, and sexual satisfaction. The independent variable was gender. Preliminary assumptions testing was conducted, and the assumptions were met. As Table 1 shows, there was no significant difference between cis women and cis men on the combined dependent variables. 

### 3.2. Central Tendency and Correlations among Variables

Table 2 contains descriptive data for the cis women’s sample, including mean, standard deviation, median, range of responses, skewness and kurtosis (normality test) for the GBD, BACDS, and a single-item measure of sexual satisfaction. Similarly, Table 3 contains the same content for the cis men’s sample. 

Regarding correlations, in the cis women’s sample, results showed that the relationship between body dissatisfaction (as measured by GBD) was significant, positive, and strongly correlated with cognitive distraction based on body appearance during sexual activity (as measured by BACDS) (*r* = 0.56; *p* < 0.001). Cis women with higher negative perceptions, behaviours, and feelings toward their body levels are more likely to experience cognitive distraction based on body appearance during sexual activity. The correlation between body dissatisfaction and sexual satisfaction was positive but nonsignificant (*r* = 0.09; *p* = 0.487). Cognitive distraction based on body appearance during sexual activity was also positive but nonsignificant correlated with sexual satisfaction (*r* = 0.11; *p* = 0.376). The assumptions for mediation [29] were not fulfilled because there was no linear relationship between the independent variable and the dependent variable and between the moderator and the dependent variable. 

Regarding correlations in the cis men’s sample, results showed that body dissatisfaction significantly, positively, and strongly correlates with cognitive distraction based on body appearance during sexual activity (*r* = 0.57; *p* < 0.001). Cognitive distraction based on body appearance during sexual activity revealed a significant, positive, and weak correlation with sexual satisfaction (*r* = 0.22; *p* < 0.05). The correlation between body dissatisfaction and sexual satisfaction was significant, positive, and moderate (*r* = 0.43; *p* < 0.001). That is, higher levels of body dissatisfaction were associated with higher levels of lack of sexual satisfaction in cis men.

### 3.3. The Mediator Effect of Cognitive Distraction Based on Body Appearance between Body Dissatisfaction and Sexual Satisfaction 

Figure 1 and Figure 2 show Models 1 and 2, respectively. These models include body dissatisfaction as the independent variable, cognitive distraction as a mediator, and sexual satisfaction as the dependent variable in cis women’s and cis men’s samples.

The regression analysis conducted in the cis men’s sample showed a statistically significant model. The total effects were significant before introducing the mediator variable. Body dissatisfaction significantly predicted sexual satisfaction, β = 0.10, 95% CI [0.01, 0.19], t = 2.16, *p* = 0.03. After introducing the mediator variable, the model presented nonsignificant direct effects, β = 0.12, 95% CI [−0.13, 0.08], t = −0.45, *p* = 0.66. These results suggest that cognitive distraction based on body appearance during sexual activity mediated the relationship between body dissatisfaction and sexual satisfaction in the cis men’s sample. Furthermore, the indirect effect of body dissatisfaction through cognitive distraction based on body appearance during sexual activity on sexual satisfaction was significant since the confidence interval does not contain 0, β = 0.10, 95% CI [0.20, 0.21].

## 4. Discussion

This study aimed to contribute new scientific and clinically relevant insights about sexual satisfaction in LGB+ people. Specifically, the study examined how cognitive distraction based on body appearance during sexual activity may impact the association between body dissatisfaction and sexual satisfaction.

Our study is one of the few that compares the sexual satisfaction levels of LGB+ cis women and cis men. Our findings show no significant differences in sexual satisfaction levels between genders. Regarding body dissatisfaction levels and cognitive distraction with body appearance during sexual activity, the results also showed no differences between cis women and cis men. These hypotheses align with the gender similarities hypothesis, suggesting that psychological variables and behaviors between genders show more similarities than differences [49]. Furthermore, it is anticipated that any observed gender differences in this domain may be influenced by contextual factors such as societal norms and gender roles rather than inherent distinctions between women and men [50]. In other words, there is the possibility that gender equality levels within different cultural settings could play a role in shaping the extent of these psychological gender differences. Since most current study participants live in Portugal—a country with laws to protect gender equality [51]—the gender differences between women and men may be small and do not translate into statistically meaningful differences in research based on self-perception measures. Furthermore, studies have shown that body dissatisfaction has become normalized for women and men, although its negative effects seem more pronounced in women [52]. That is, the normalization of body dissatisfaction suggests that being unhappy with one’s body has become a widespread phenomenon that is unquestionably accepted in Western society. Research has shown that body dissatisfaction can lead to cognitive distraction during sexual activity among heterosexual people [16], indicating a high prevalence of body dissatisfaction. However, our study did not find such a result. We speculate that sexual orientation may act as a protective factor in this regard. 

It is important to note that in the cis women’s sample, there is a correlation between body dissatisfaction and cognitive distraction related to body appearance during sexual activity. Still, neither of these factors is associated with sexual satisfaction. These findings may indicate that the factors linked to the sexual satisfaction of LGB+ cis women do not appear to be related to body issues. Unexpectedly, this result is not in line with another study [30], which states that the sexual satisfaction of LGB+ people is also influenced by factors shared with heterosexual people (such as body image). Our study may suggest that for cis women who self-identify as LGB+, sexual orientation may protect against the negative impacts of body dissatisfaction often found in heterosexual women [29]. This result is in line with research demonstrating that LGB+ cis women, predominantly lesbian cis women, may not feel pressured to meet the standards of beauty based on male preferences in a heteronormative society [53]. That way, in LGB+ cis women, body image may have a reduced impact on their sexual satisfaction compared to heterosexual peers. Other factors may be more significant in determining sexual satisfaction among LGB+ cis women. We recommend that further studies adopt a developmental perspective and investigate what factors explain the lower vulnerability to body dissatisfaction in LGB+ cis women and how they evolve throughout different life phases. 

Regarding the correlation between body dissatisfaction, body appearance cognitive distraction, and sexual satisfaction in the cis men’s sample, the correlation was significant and negative and met all the assumptions to test a mediation model that proved to be significant, confirming that LGB+ cis men who experience body dissatisfaction may experience increased cognitive distraction during sexual activity and reduced sexual satisfaction [54]. Our results are in line and are supported by studies indicating that the gay male subculture places great importance on physical appearance, which can lead to a perceived gap between their actual body image and their desired body shape [55]. This gay subculture often promotes the idea that self-worth is tied to a specific aesthetic [56]. Research suggests that gay men base their worth on physical appearance more than heterosexual people [57]. In other words, while messages from the dominant culture may not prompt heterosexual men to evaluate themselves aesthetically, the gay community may have different messages that influence body image perceptions. This is supported by a recent systematic review [58] that shows that striving for bodily perfection can be a response to the harassment, discrimination, and perceived inferiority that gay men deal with. In other words, the intersection of body image ideals, minority stressors, and cultural expectations could influence the negative correlation between body dissatisfaction and sexual satisfaction. According to this, these intersections may translate into more vulnerability to being cognitively distracted about their body in sexual contexts and have poorer sexual outcomes, namely, less sexual satisfaction. Indeed, in a recent study involving a sample of gay and bisexual men [59], the findings underscored the persistent impact of stigma, which extends to various aspects of life, including sexual satisfaction. The consequences of stigma were associated with the ageing process. As men grow older, the detrimental effects of body dissatisfaction on the well-being and sexual satisfaction of gay and bisexual men may become more pronounced.

In a simplified way, both LGB+ cis women and LGB+ cis men report body dissatisfaction, but for LGB+ cis women, this may not relate to their perception of their sexual value.

Taken together, the findings support the validity of the cognitive models (Barlow’s [19] and Nobre’s [20,21]). Our statistical and conceptual model was only significant for cis men, which reinforces the socio-cognitive nature of the cognitive approach and that they should be applied considering the social context.

This study emphasizes the significance of researchers, sex educators, and clinicians in understanding how cultural ideals of attractiveness, body monitoring, and shame can affect people. Educating people on how societal pressures may manipulate perceptions would help them deal with these detrimental widespread standards. It can also enable people to differentiate between body monitoring efforts that stem from societal norms and those based on legitimate safety concerns, which can help reduce harmful body vigilance and shame. It is also important that clinicians consider the impact that minority stressors have on the experiences of LGB+ people in relationships. A recent study demonstrates that the discrimination felt by LGB+ people starts from an early age, highlighting that adolescents in committed same-sex relationships in Portugal already showed moderate levels of LGB+ oppression, resulting in poorer relationship satisfaction [60]. By doing so, clinicians can promote the sexual satisfaction and empowerment of LGB+ people. Furthermore, in clinical contexts, strategies such as cognitive restructuring should be employed to challenge the appearance beliefs of people. Additionally, our findings may emphasize the importance of promoting clinical skills, such as mindfulness, in refocusing patients’ attention on erotic cues. Prevention programs and evaluation of problems related to sexuality must consider these variables. 

### 4.1. Limitations 

This study has several limitations. It was a cross-sectional study, so we cannot determine causality and mediation is based on theory. Additionally, the sample size of LGB+ cis people was small and non-representative, which may limit the generalizability of the results. We also did not conduct analysis based on sexual orientation, so we may not have captured all the nuances of LGB+ peoples’ experiences. Furthermore, the measures were acquired exclusively through self-report questionnaires, and it would be beneficial to incorporate other assessment methods. 

### 4.2. Future Lines of Research

Further studies should replicate these analyses and consider whether bisexual people were involved in same-sex or other-sex relationships. It is also recommended that research studies be conducted with a more heterogeneous group of people with different sexual orientations. Studies with black, indigenous, people of color (BIPOC), and other marginalized groups (e.g., people with functional diversity) are also necessary. This will allow for a more comprehensive and inclusive analysis of the subject matter.

Future studies should also look at specific aspects of sexual satisfaction (e.g., orgasm, communication) to better understand how body dissatisfaction plays a role in LGB+ people’s experiences. Lastly, we only included cisgender people, so future studies should examine the impact of body image on trans people. We can gain a more comprehensive understanding of this topic by addressing these limitations. 

Despite the study’s limitations, it offers valuable insights that enhance previous research and provides a deeper understanding of sexual satisfaction and the suitability of cognitive models of sexual response in LGB+ people, pushing the boundaries of knowledge in this area. Future studies within this model could integrate other theoretically relevant trait-like cognitive variables, such as sexual attitudes [61] and beliefs [62], to better determine the extent of the influence of these variables on sexual outcomes.

## 5. Conclusions

This study sheds light on the relationship between body dissatisfaction, cognitive distraction based on body appearance during sexual activity, and sexual satisfaction in LGB+ cis people. It reaffirms the significance of cognitive psychology models in explaining sexual response. While gender similarities prevail in levels of body dissatisfaction and body appearance cognitive distraction, the differential impact on sexual satisfaction is evident. LGB+ cis women appear less affected by body dissatisfaction, possibly due to not needing to correspond to societal expectations, which may be associated with positive sexual outcomes. On the other hand, cis men who identify as LGB+ are more likely to experience an association between body dissatisfaction and reduced sexual satisfaction. This may be due to the emphasis on appearance within the gay male subculture. These findings highlight the importance of healthcare, education, and policy-maker professionals considering cultural ideals and their impact when addressing body image and sexual well-being concerns in LGB+ cis people regardless of their sexual function and clinical status.

## Figures and Tables

**Table 1:** MANOVA analysis to investigate differences between cis women and cis men in the dependent variables.

	F (3,159)	*p*	Wilks’ Lambda	η_p_^2^
Cis women * Cis men	0.40	0.755	0.99	0.05

**Table 2:** Mean, *SD*, median, and range of body dissatisfaction, cognitive distraction based on body appearance, and sexual satisfaction in cis women.

	Mean	*SD*	Median	Range	Skewness	Kurtosis
Body dissatisfaction	6.54	3.93	6.00	0–18	0.95	0.75
Cognitive Distraction	16.25	8.01	13.00	10–43	1.80	3.10
Sexual Satisfaction	1.77	1.59	1.00	1–6	1.79	1.68

**Table 3:** Mean, *SD*, median, and range of body dissatisfaction, cognitive distraction based on body appearance, and sexual satisfaction in cis men.

	Mean	*SD*	Median	Range	Skewness	Kurtosis
Body dissatisfaction	6.37	3.48	6.00	0–19	1.03	1.48
Cognitive Distraction	15.61	6.60	14.00	10–41	1.94	4.14
Sexual Satisfaction	1.96	1.61	1.00	1–6	1.27	−0.08

## Data Availability

The data presented in this study can be available upon request from the corresponding author. The data are not publicly available due to ethical and privacy restrictions.
